# Editorial: Nutriomic analysis of food and functional compounds for MAFLD prevention

**DOI:** 10.3389/fnut.2025.1666502

**Published:** 2025-08-05

**Authors:** Fermin I. Milagro, Claudio A. Bernal

**Affiliations:** ^1^Department of Nutrition, Food Science and Physiology, Faculty of Pharmacy and Nutrition, University of Navarra, Pamplona, Spain; ^2^Navarra Institute for Health Research (IdiSNA), Pamplona, Spain; ^3^Centro de Investigación Biomédica en Red de la Fisiopatología de la Obesidad y Nutrición (CIBEROBN), Instituto de Salud Carlos III, Madrid, Spain; ^4^Red Iberoamericana de Colaboración Académica y Científica en Nutriómicas y Nutrición de Precisión RINN22, Monterrey, Mexico; ^5^Departamento de Ciencias Biológicas, Bromatología y Nutrición, Facultad de Bioquímica y Ciencias Biológicas, Universidad Nacional del Litoral, Santa Fe, Argentina; ^6^Consejo Nacional de Ciencia y Técnica (CONICET), Santa Fe, Argentina

**Keywords:** microbiota, vitamin D, sodium, NAFLD, MASLD, MAFLD, inflammation, ultra-processed food

This Research Topic aims to collect manuscripts that deepen our understanding of metabolic-associated fatty liver disease (MAFLD), with particular emphasis on elucidating the nutriomic effects of foods and functional compounds in the prevention of MAFLD and its associated clinical outcomes. It is important to note that MAFLD was previously referred to as non-alcoholic fatty liver disease (NAFLD), and recently (1), a new nomenclature has been proposed: metabolic dysfunction-associated steatotic liver disease (MASLD), with all the related conditions encompassed under the broader term “steatotic liver disease” (SLD). MAFLD is currently the most common chronic liver disease, and its global incidence is rising at an alarming rate. Approximately 30% of the adult population worldwide is estimated to be affected, although prevalence rates vary considerable between regions or countries (2). Importantly, the progression, and eventually the regression, of MAFLD appears to be regulated by nutrigenomic and epigenomic factors that represent new therapeutic targets for dietary and lifestyles interventions (3, 4). Therefore, in this Research Topic there are 6 contributions that explore how dietary fatty acids, vitamin D, sodium intake, ultra-processed foods (UPF), gut microbiota, and indexes of inflammation and dietary quality influence the pathogenesis and potential reversal of MASLD/MAFLD.

In the initial manuscript of this Research Topic, Chen et al., based on a large-scale retrospective case-control study (2,470 adults) using data collected in 2017–2018 from the National Health and Nutrition Examination Survey (NHANES), shows that dietary fatty acids (FA), except for polyunsaturated FA (PUFA), may increase the risk of developing NAFLD, particularly in obese individuals aged 37–54 years. Specifically, the authors explored the role of the sex in NAFLD development and found that the ratios of unsaturated FA (UFA)/saturated FA (SFA), PUFA/SFA, monounsaturated FA (MUFA)/SFA, and PUFA/MUFA were associated with NAFLD risk in males, with higher ratios predicting a lower risk. Interestingly, increasing the proportion of unsaturated FA in the diet, especially PUFA, appears to be a promising strategy for preventing NAFLD in middle-aged obese men. Zhang et al. carried out a bibliometric and visual analysis of 359 publications obtained between 2007 and 2024 that explore the link between vitamin D (VD) and metabolism-associated fatty liver disease (MAFLD), mapping the research landscape, trends, hotspots, and emerging frontiers to guide future investigations. Although it is well-established that VD deficiency is associated with increased prevalence of obesity and MAFLD, the effectiveness of VD supplementation in improving MAFLD outcomes remains controversial and requires further investigation. Li et al. throughout a longitudinal cohort study analyzed the relationship between sodium intake and the incidence and all-cause mortality of NAFLD. The analysis included data from 13,853 American adults by comprehensively reviewing the literature and analyzing data from The National Health and Nutrition Examination Survey (NHANES, 2003–2018), including 4,465 participants with NAFLD. The findings suggest that higher sodium intake in individuals with NAFLD is associated with increased disease incidence but decreased all-cause mortality. Notably, the dose–response relationship between sodium intake and mortality risk exhibited a non-linear pattern, with a critical inflection point around 3.5 grams per day. Following the manuscripts addressing the interaction between nutrients and MAFLD, Shi et al. have studied the impact of UPF on liver health in rats and explored the role of gut microbiota and metabolites. They have observed that UPF increased potentially harmful bacteria in the gut microbiota and led to a metabolomic disorder characterized by disruptions in the sphingolipid signaling pathway, sulfur relay system, and arachidonic acid metabolism. Sang et al. explored the association between the Dietary Inflammation Index (DII) and other indicators of liver disease severity like hepatic fibrosis and the Fatty Liver Index (FLI) within a cohort of U.S. adults diagnosed with MASLD. They describe that no meaningful statistical relationship exists between DII scores and the risk of liver fibrosis or increased FLI and emphasizes that dietary factors should be carefully considered in the clinical evaluation of disease progression in patients with MASLD. Finally, Huang et al. explored the influence of five dietary quality indexes on mortality among NAFLD patients and advanced fibrosis patients, and discovered that a high-quality diet could potentially mitigate mortality risk in both groups of patients. These results reinforce the idea that nutritional strategies may serve as an effective, non-pharmacological approach to reducing mortality rates and improving outcomes.

As a conclusion, the studies included in this Research Topic provide valuable insights into the comprehension of MAFLD and its associated comorbidities, highlighting the role of foods, nutrients and gut microbiota. The conclusions presented in this Research Topic may serve as a useful reference for both the prevention and treatment of MAFLD, as well as offer meaningful guidance for future research. However, the manuscripts published in this Research Topic also underscore that many aspects of the complex interplay between nutrition and MAFLD remain to be fully elucidated, and new additions can be added to the consensus statements that currently lay the foundation for customized dietary guidelines (5).
